# Topical application of cannabidiol for muscle recovery after exercise-induced muscle damage: a randomized, double-blinded pilot study

**DOI:** 10.1186/s42238-026-00420-0

**Published:** 2026-03-25

**Authors:** Antoine Mendes, Gregory Cuvelier, Agathe Anthierens, Appoline Locquenies, Nathan Gauchet, Patrice Maboudou, Elsa Heyman, Serge Berthoin, François-Xavier Gamelin

**Affiliations:** 1 Univ Lille, Univ Artois, Univ Littoral Côte d’Opale, ULR 7369 - URePSSS - Unité de Recherche Pluridisciplinaire Sport Santé Société, Lille, F-59000 France; 2 Laboratoire de L’Effort Et du Mouvement, HEPH-Condorcet, Tournai, Belgique; 3 CHU Lille, Service de Biochimie Automatisée Protéines, Lille, F-59000 France; 4 Institut Universitaire de France, (IUF), Paris, F-75005 France

**Keywords:** Cannabis, CBD, Delayed onset muscle soreness, Isokinetic, Recovery

## Abstract

**Introduction:**

This study investigated the effects of a topical cannabidiol (CBD) gel compared to placebo on muscle function recovery, perceived muscle soreness, and blood marker of muscle damage following strenuous exercise designed to induce muscle damage.

**Methods:**

Fifteen physically active students (age: 21.4 ± 2.1 years; mass: 78.0 ± 9.5 kg; height: 181.7 ± 5.0 cm) participated in the study. To induce muscle damage, participants completed 10 sets of 10 drop jumps from a 0.6 m box, with 2 min of rest between sets. In a randomized crossover design, 2 g of either CBD gel or placebo gel were applied to the quadriceps and hamstrings immediately after exercise and over the following 72 h. Muscle function recovery was assessed by measuring isometric and concentric isokinetic peak torque of the knee extensors, plasma myoglobin (Myo) concentration, and Delayed Onset Muscle Soreness (DOMS), evaluated using a visual analogue scale (VAS) at baseline, immediately post-exercise, and at 24-, 48-, and 72-h intervals.

**Results:**

The two-way ANOVA revealed no significant interaction between time and condition for concentric isokinetic peak torque (*p* > 0.05). No significant differences were observed between the CBD and placebo conditions for plasma Myo concentrations or DOMS scores. A significant interaction was observed for isometric torque (*p* = 0.02), with moderate effect sizes favoring CBD over placebo from 24 to 72 h post-exercise (ES = 0.85–1.0).

**Conclusion:**

The topical application of CBD gel does not appear to accelerate the recovery of muscle function or to reduce DOMS following muscle-damaging exercise.

**Ethical approval number:**

B200-2020–120.

**Supplementary Information:**

The online version contains supplementary material available at 10.1186/s42238-026-00420-0.

## Introduction

Cannabidiol (CBD) and Δ9-tetrahydrocannabinol (Δ9-THC) are the most well-known phytocannabinoids found in the cannabis plant (Corroon and Phillips 2018). Unlike Δ9-THC, CBD is non-intoxicating and possesses pharmacological properties of interest for medical applications (Gamelin et al. 2020). In 2018 and 2019, the U.S. Food and Drug Administration (FDA) and the European Medicines Agency (EMA), respectively, approved Epidiolex, an oral CBD solution for the treatment of rare and severe forms of pediatric epilepsy (U.S.Food Drug Administration 2018; European Medicines Agency 2023). Beyond this medical indication and depending on local legislation, CBD is also commercially available in various forms, including oils, sprays, pills, tinctures, e-liquids, and balms for oral or topical administration (Iffland and Grotenhermen 2017). Its growing popularity is likely driven by preclinical and clinical evidence suggesting anxiolytic, anti-inflammatory, analgesic, and neuroprotective effects, along with a relatively favorable safety profile (Iffland and Grotenhermen 2017; Moltke and Hindocha 2021). Among the general population, the most commonly reported reasons for CBD use include managing anxiety, stress, and sleep disturbances (Moltke and Hindocha 2021).

Since the World Anti-Doping Agency (WADA) removed CBD from its list of prohibited substances in 2018, and in light of its pharmacological properties, this cannabis-derived compound has attracted considerable attention from athletes (Gamelin et al. 2020; Kasper et al. 2020). Despite the possibility of a positive anti-doping test due to product contamination with other cannabinoids (such as Δ9-THC), which remain prohibited by WADA, athletes appear to be using CBD-based products. Kasper et al. (2020) reported a 28% prevalence of CBD use among professional rugby players. Among users, the main motivations were pain relief and enhanced recovery. Competitive sport and training can result in muscle damage, tissue inflammation, delayed-onset muscle soreness (DOMS), and a temporary decrease in muscle function, all of which is associated with an increased risk of injury (Dupuy et al. 2018). In this context, CBD has emerged as a promising candidate for improving recovery. A growing volume of research has investigated CBD’s potential analgesic and anti-inflammatory effects on recovery, although most of this research has been conducted in preclinical settings or clinical trials outside the field of sports science (Gamelin et al. 2020; Schouten et al. 2022; Rojas-Valverde 2021).

Despite its growing popularity, few clinical trials have directly assessed the effectiveness of CBD for recovery following intense exercise. After an intense aerobic exercise, 50 mg or 300 mg oral intake of CBD does not appear to influence the short-term recovery of blood markers of muscle damage (Creatine kinase (CK) and Myoglobin (Myo)) (Sahinovic et al. 2025). In contrast, 72 h after a resistance exercises, Isenmann et al. (2021) reported a significant positive effect of orally administered CBD (60 mg) on CK and Myo, also as well as improved squat performance. They reported also an improvement in Myo levels after a 6-day period of intense training in advanced athletes supplemented daily with 60 mg of CBD compared to highly trained athletes (Isenmann et al. 2024). However, other studies have not found significant effects of CBD on muscle function, inflammatory markers (*i.e.,* Interleukins 6,and 10), muscle damage (*i.e.,*Myo), or perceived soreness in the days following intense eccentric exercise (Crossland et al. 2022; Cochrane-Snyman 2021). It is likely that the effectiveness of CBD during recovery depends on both the type of exercise and the training status of the individual using it.

The studies cited above primarily investigated oral CBD supplementation (Isenmann et al. 2021, 2024; Crossland et al. 2022; Cochrane-Snyman 2021) however, CBD can also be administered topically via application to the skin. Although this method is less commonly used among CBD users (Corroon and Phillips 2018), it has demonstrated effectiveness in treating different disorders (Crossland et al. 2022; Cochrane-Snyman 2021). Topical or transdermal application of CBD has demonstrated efficacy in various types of pain in humans with CBD concentrations around 20–30 mg per day, such as osteoarthritis-related pain (Bawa et al. 2024) myofascial pain (Walczyńska-Dragon et al. 2025) or chronic pain resulting from acute lower extremity injuries (Hall et al. 2023). This route of administration may be interesting means to avoid the first- pass metabolism that limits oral bioavailability of CBD (~ 6%) (Martinez Naya et al. 2024). Moreover, given that repeated oral intake of CBD over short periods (6 days) is known to potentially alter liver activity (Isenmann et al. 2024), it would be interesting to explore the possibility of a more localized mode of action. For instance, when using nonsteroidal anti-inflammatory drugs, this administration strategy minimizes systemic exposure while enhancing local drug concentrations (Rolf et al. 1999; Kienzler et al. 2010).

To our knowledge, only two studies have investigated the effects of topical CBD application following exercise-induced reductions in muscle function (Alpy 2023; Pastina 2024). These studies found no significant effect on the recovery kinetics of muscle function or muscle soreness. However, they did not assess indirect blood markers of muscle damage such as CK and Myo which provide valuable insights into the biological impact of exercise.

**Fig. 1:**
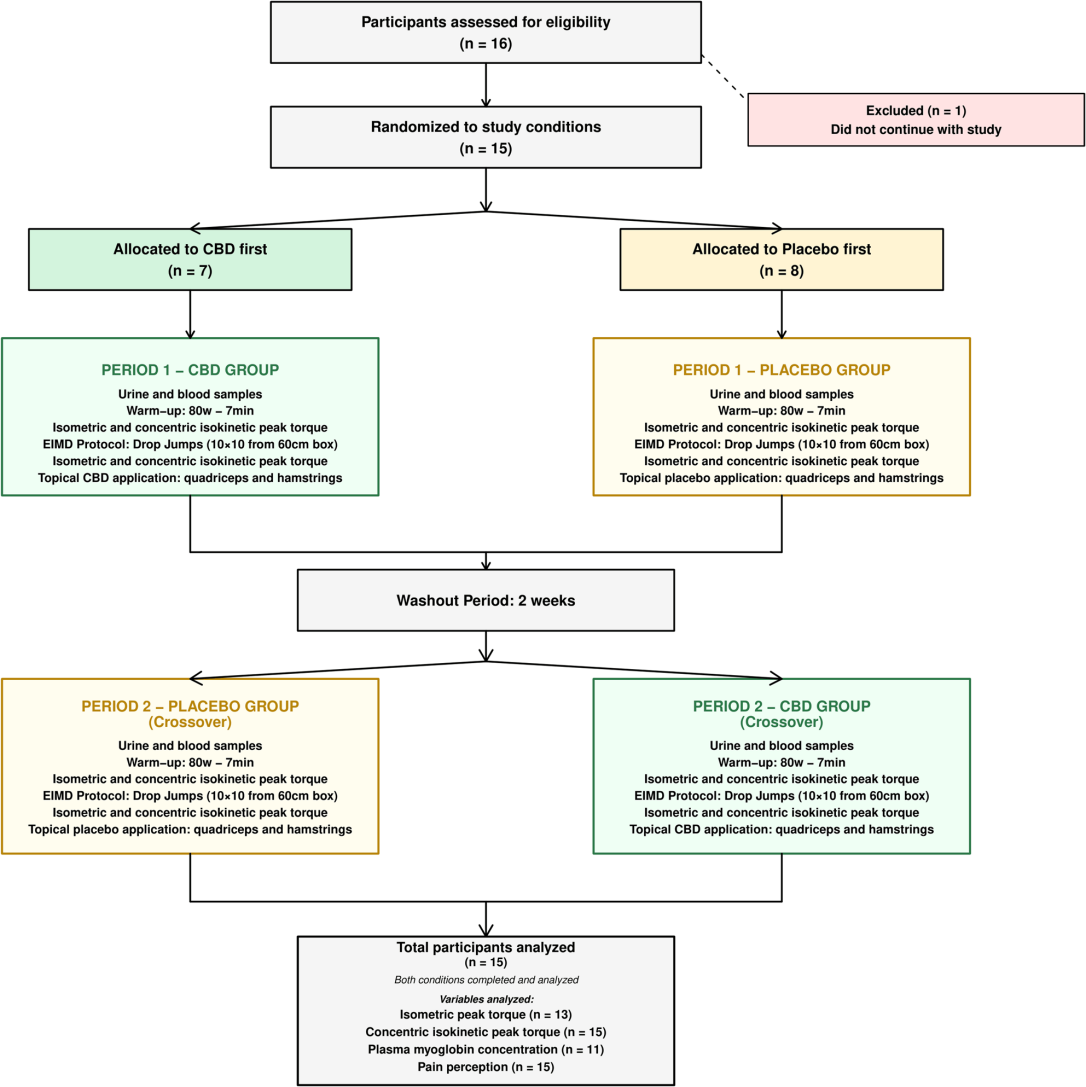
Flow diagram of participant progress through the randomized cross-over CBD vs placebo trial

Therefore, the aim of this study was to investigate the effects of a CBD gel on muscle function, DOMS, and blood marker of muscle damage following strenuous exercise designed to induce muscle damage. We hypothesized that the experimental group receiving CBD would experience faster recovery than the placebo group, due to the potential effects of CBD on recovery-related mechanisms Fig. 1.

## Methods

### Subjects

Sixteen participants were screened for eligibility, and one withdrew before completing the study. Finally, 15 physically active male physiotherapy students (engaging in > 3 × 30 min of physical activity per week) participated in the study (age: 21.4 ± 2.1 years; body mass: 78.0 ± 9.5 kg; height: 181.7 ± 5.0 cm). All participants provided written informed consent. None were regular users of cannabis or hemp-derived products, and none had used such products in the previous six months. Participants were instructed to refrain from any physical exercise for 48 h, and from alcohol and caffeine for 24 h, prior to the first visit and throughout the protocol. All participants were already familiar with isokinetic testing as part of their physiotherapy training. The study protocol complied with the guidelines of the Declaration of Helsinki (revised in 2024) and was approved by the Academic Ethical Committee Brussels Alliance for Research and Higher Education (Belgium), approval number B200-2020–120. The study was also conducted in accordance with the CONSORT (Consolidated Standards of Reporting Trials) guidelines for randomized trials, and the checklist was carefully followed to ensure transparency and methodological rigor.

### Procedure

A double-blinded, randomized, balanced crossover design was used to investigate the effect of a CBD gel on muscle function recovery at 24, 48, and 72 h following an exercise protocol intended to induce muscle damage. The order of the experimental conditions (CBD or placebo) was randomized for each participant using a simple drawing of lots prior to the start of the study. Briefly, a total of sixteen treatment sets were assigned to participants. These sets were preassembled by the gel manufacturer (Becann Company, France) and contained the products to be applied in chronological order. Eight sets-initiated treatment with the CBD gel followed by the placebo, whereas the remaining eight sets began with the placebo gel followed by the CBD condition. This procedure was implemented in order to have approximately half of the participants start with the CBD condition and the other half with the placebo condition. In our study, blinding was maintained after intervention allocation for several roles. Participants were unaware of whether they received the CBD gel or the placebo. The investigators who applied the gels, supervised the sessions, performed the outcome assessments (muscle function tests, blood analyses, soreness evaluations), and processed the data were all blinded to the intervention assignment. Data were coded anonymously until final interpretation. This double-blinding procedure was rigorously upheld throughout the study to minimize bias. The study comprised two randomized phases (CBD vs. Placebo), each lasting 4 days, and separated by a 2-week washout period.

During the first laboratory visit, participants were weighed and measured, and a venous blood sample was collected to analyze plasma Myo levels. After a 7-min warm-up at an intensity of 80 watts on a cycle ergometer (E894, Monark), isometric peak torque (at 60°) and concentric isokinetic peak torque (at 60°·s⁻¹) of the knee extensors were measured using an isokinetic dynamometer. Subjective muscle soreness in the exercised leg was assessed using a VAS during the concentric isokinetic peak torque test. After a 2-min recovery period, participants completed the muscle damage-inducing exercise protocol. New measurements of isometric and concentric isokinetic torque and perceived muscle soreness were then collected, after which participants were randomly assigned to receive either a CBD-containing gel (1% CBD) or a placebo gel (0% CBD). The same gel was used throughout the entire phase, and neither participants nor experimenters knew the gel’s composition. Two grams of gel (containing 20 mg of CBD or placebo) were massaged into the quadriceps and hamstring muscles of the participant’s dominant leg for 1 min per muscle group. Participants were instructed not to wash their legs for the following 6 h. No familiarization session for isokinetic strength assessment was conducted before the protocol as the participants, being physiotherapy students, were regularly exposed to this type of evaluation during their training.

At 24-, 48-, and 72-h post-exercise, participants returned to the laboratory under the same conditions as during the first visit. At each time point, blood samples were collected, and isometric peak torque, concentric isokinetic peak torque, and perceived muscle soreness were assessed. At 24 and 48 h, the dominant leg was again massaged with 2 g of gel for 1 min per muscle group, 2 min after the isokinetic test. Participants were instructed not to wash their legs for the following 6 h.

During the second phase, following the 2-week washout period, participants completed the same protocol using the alternate gel (i.e., CBD or placebo). Throughout the two-week assessment period, subjects were also instructed not to perform any muscular activity involving leg muscles or any strength training for any body part during the experimental period. Additionally, they were instructed to maintain the same diet throughout the two-week testing period and not to eat protein before or after the recovery sessions or use other recovery strategies.

### Isometric and concentric isokinetic peak torque

Isometric peak torque of the dominant leg was measured during a maximal voluntary contraction using a dynamometer (Cybex II, Biodex, USA) in accordance with Abaidia et al. (Abaïdia et al. 2017). After calibrating the device, participants were seated in an upright position with the hip joint at 75°. Full extension of the leg was considered as 0° for dynamic tests (range of motion 0–90°). The distal shin pad of the dynamometer was attached 3–4 cm proximal to the lateral malleolus by using a strap. The thigh was secured to the seat with a Velcro strap, and the ankle was fastened to the lever arm with an ankle pad. The dynamometer’s axis of rotation was aligned with the lateral femoral condyle, and the knee joint angle was set at 60° of flexion. Participants performed three 5-s maximal contractions, with 1 min of rest between each attempt. Following a 3-min recovery period, concentric isokinetic peak torque was measured on the same leg. Participants performed three trials, each consisting of three successive maximal concentric knee extensions at an angular velocity of 60°·s⁻¹, with 3-min rest intervals between trials. Experimenters provided verbal encouragement during all maximal voluntary contractions. For both isometric and concentric isokinetic peak torques, the best performance was retained for analysis. Due to technical issues with the dynamometer, isometric performance data were unavailable for two participants.

### Exercise-induced muscle damage protocol

The muscle-damaging protocol was adapted from Ferreira-Junior et al. (2015). It consisted of 100 drop jumps from a 0.6-m box, 10 sets of 10 jumps with 2 min of passive recovery between each set. Upon landing, subjects were instructed to flex their knees to at least 90° (0° = full extension) and then immediately jump as high and explosively as possible, using an arm swing to maximize jump height. After each jump, they stepped back onto the box. During recovery periods, participants remained seated on a chair. Verbal encouragement was provided throughout the exercise.

### Gel

The gels were aqueous formulations containing a synthetic polymer (CARBOPOL 980), with 1% CBD in the experimental condition and 0% CBD in the placebo condition. Both gels were identical in color, smell, and texture, and were presented in identical containers. Each gram of gel contained 10 mg of CBD. A total of two grams of gel were applied, with one gram massaged onto the skin over each of the quadriceps and hamstring muscles by the experimenter using a vinyl glove. A 250 cm^2^ area was marked on each muscle group with a permanent marker to ensure consistent application sites across visits. The gel was applied using a circular massage motion for 1 min. The gels were supplied by Becann Company (France).

### Blood collection

At each visit, following a 10-min rest in a seated position, a venous blood sample was collected from an antecubital vein to determine plasma Myo concentration. Blood was drawn into a 9 mL EDTA tube, immediately centrifuged at 3000 rpm for 5 min at 4 °C, then aliquoted and stored at −80 °C. Plasma Myo concentrations were measured by nephelometry using a BNII analyzer (Siemens Healthcare Diagnostics). Due to technical issues, plasma analysis was completed for only 11 participants.

### Visual analogue scale pain scores

Delayed Onset Muscle Soreness was assessed using a 10 cm VAS, where 0 cm represented “no pain” and 10 cm represented “the worst imaginable pain.” Participants were instructed to mark a cross on the line corresponding to their perceived level of muscle soreness. This assessment was conducted at five time points: baseline (prior to exercise), immediately post-exercise, and at 24-, 48-, and 72-h post-exercise, providing a standardized measure of temporal changes in perceived muscle soreness following the intervention.

### Statistical analysis

Data are presented as means ± standard deviation (SD). Due to technical issues encountered with certain parameters during data collection, the number of participants (n) varied across some statistical analyses. The Shapiro–Wilk test was used to assess normality, and homoscedasticity was verified using a modified Levene’s test. Because of heteroscedasticity, plasma Myo data were log-transformed prior to analysis. A two-way repeated measures ANOVA was used to evaluate the effects of condition (placebo vs CBD), time, and the condition-by-time interaction on isometric peak torque (*n* = 13), concentric isokinetic peak torque (60°·s⁻¹) (*n* = 15), plasma Myo levels (*n* = 11), and VAS scores (*n*= 15). Post-hoc Bonferroni tests were conducted for multiple comparisons when significant main effects or interactions were observed. Effect sizes (ES), calculated as the mean difference divided by the pooled variance (Cohen’s d) with 95% confidence intervals (CI), were used to assess the magnitude of differences between conditions (placebo vs CBD) from baseline percentages. Cohen's d values were interpreted as trivial (< 0.2), small (0.2–0.59), moderate (0.6–1.19), large (1.2–1.9) or very large (Gamelin et al. 2020; U.S. 2018; epidyolex-epar-public-assessment-report_en.pdf. 2023) according to Hopkins et al. (2009). Statistical significance was set at*p* < *0.05* for all analyses. All statistical analyses were conducted using Statistica 12.0 software.

## Results

**Fig. 2:**
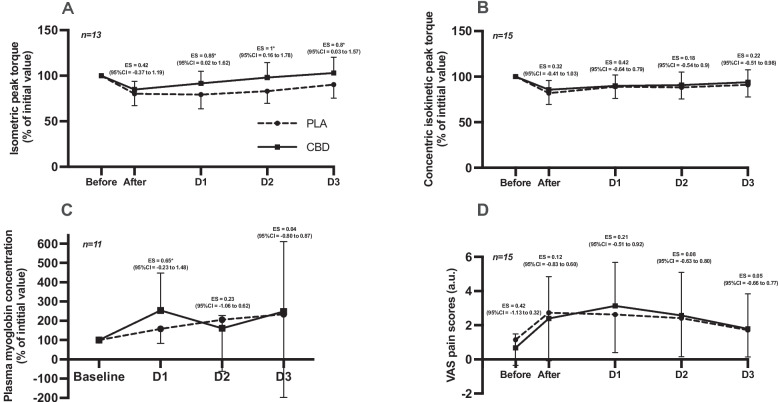
Kinetics of muscle function, plasma myoglobin and perceived muscle soreness. **A**: Isometric peak torque of knee extension at 60° expressed as a percentage of initial value. **B**: Concentric isokinetic peak torque of knee extension at 60°·s⁻¹ expressed as a percentage of initial value. **C**: Plasma myoglobin concentration expressed as a percentage of initial value. **D**: Perceived muscle soreness assessed by VAS, expressed in arbitrary units (a.u.). PLA: Placebo; CBD: Cannabidiol; ES: Effect size (Cohens’d); CI: confidence interval

Results of the two-way ANOVA for the interaction (Condition and Time) and main effects (Condition, Time) on muscle function parameters, plasma Myo, and VAS scores are presented in Table 1. The two-way ANOVA for isometric peak torque (*n* = *13)* revealed a significant main effect of time (see Table 1), as well as a significant Condition and Time interaction. In the CBD condition, isometric peak torque significantly decreased (*p* < 0.001) immediately after exercise but returned to baseline after 24 h of recovery. In contrast, in the placebo condition, isometric peak torque also decreased significantly post-exercise (*p* < 0.0001) and remained significantly below baseline at both 24 and 48 h of recovery (*p* < 0.0001 for both). From 24 h onward, the difference in isometric peak torque between the CBD and placebo conditions, expressed as a percentage of initial value, showed a moderate effect size (ES = 0.85). This moderate effect persisted at 48- and 72-h post-exercise (ES = 1.0 and 0.8, respectively; Fig. 2A). For concentric isokinetic peak torque (*n* = *15*), two-way ANOVA showed a significant main effect of time (Table 1), with no significant effect of condition or Condition and Time interaction. Differences in concentric isokinetic peak torque between the CBD and placebo conditions, expressed as a percentage of baseline, are presented in Fig. 2B. These differences were trivial immediately after exercise and at 72 h, with only small effects observed at 24- and 48-h post-exercise. For plasma Myo (*n* = *11*), two-way ANOVA indicated a significant main effect of time (Table 1), but no significant effect of condition or Condition and Time interaction. Plasma Myo concentrations, expressed as a percentage of baseline, are illustrated in Fig. 2C. At 24 h post-exercise, the effect size between CBD and placebo conditions was moderate (ES = 0.65) but this effect decreased at 48 and 72 h. VAS scores (*n* = *15*) revealed a significant main effect of time, with no significant condition effects or Condition and Time interaction (Table 1). Figure 2D illustrates the magnitude of differences in perceived muscle soreness between the CBD and placebo conditions, with no moderate or larger effect sizes observed before, immediately after, or at 24-, 48-, and 72-h post-exercise.

**Table 1:** Two-Way ANOVA outcomes for muscle function, myoglobin and DOMS Over time and condition

Parameters	Main effect ANOVA	F Values	*P Values*
Isometric peak torque 60° *(N.m)* (*n* = 13)	Cond x Time	*F*_(4, 96)_ = 3.03	0.02
	Cond	*F*_(1, 24)_ = 0.59	NS
	Time	*F*_(4, 96)_ = 19.51	0.0001
Concentric peak torque 60°.s^−1^ *(N.m)* (*n* = 15)	Cond x Time	*F*_(4, 108_) = 0.60	NS
	Cond	*F*_(1, 27)_ = 0.13	NS
	Time	*F*_(4, 108)_ = 15.72	0.0001
Concentration Myo *(Ln)* (*n* = 11)	Cond x Time	*F*_(3, 60)_ = 0.882	NS
	Cond	*F*_(1, 20)_ = 0.483	NS
	Time	*F*_(3, 60)_ = 5.21	0.0001
VAS score *(u.a)* (*n* = 15)	Cond x Time	*F*_(4, 112)_ = 0.79	NS
	Cond	*F*_(1, 28)_ = 0.0004	NS
	Time	*F*_(4, 112)_ = 12.79	0.0001

The main effects from ANOVA are as follows: *Time* time effect, *Cond* condition effect (CBD or Placebo), × interaction between variables, *NS* not significant. F-statistic and its associated degrees of freedom and *p* value of analyses of variance used to compare the effects of placebo or CBD gel on Isometric peak torque; Isokinetic concentric peak torque; concentration of plasma myoglobin; VAS score

## Discussion

This pilot study aimed to evaluate the effect of a topical application of CBD gel on muscle function recovery, a blood marker of muscle damage and DOMS, over the three days following strenuous exercise. As previously shown, this type of protocol induces muscle damage and a reduction in muscle function (Ferreira-Junior et al. 2015).

A major finding of this study is the significant Condition and Time interaction observed for isometric peak torque recovery. Specifically, isometric force levels returned to baseline within 24 h in the CBD condition, whereas recovery took 72 h in the placebo condition. However, this difference should be interpreted with caution and does not necessarily reflect a true effect of CBD. Importantly, although 15 participants were randomized, isometric peak torque analyses were performed on 13 participants because isometric data from two participants were unavailable due to technical issues. Accordingly, these two participants are not included in Fig. 2a (*n*= 13). The missing data resulted in an imbalance in favor of the CBD condition during the second session (8 vs 5, respectively). In addition, the short washout period may have allowed a repeated bout effect (RBE). RBE is an adaptive physiological response in which muscles experience reduced damage following a repeated eccentric or otherwise unfamiliar form of exercise (Calvo-Rubio et al. 2024). The crossover protocol designs helps to avoid the excessive influence of this effect if the subjects are distributed in a balanced manner. This methodological limitation provides a plausible explanation for the differences observed in the CBD condition (Supplementary Table 1). It is therefore reasonable to assume that CBD gel had no effect on the recovery of isometric peak torque. This interpretation is supported by the kinetics of concentric isokinetic peak torque, which was not affected by technical issues and did not show a comparable condition effect. For this other muscle function marker, our findings are consistent with previous studies that investigated topical CBD application following exercise-induced muscle damage (Alpy 2023; Pastina 2024). Specifically, CBD did not accelerate the recovery of concentric isokinetic torque at 24-, 48- or 72-h post-exercise. Pastina et al. (2024) reported no improvement in counter movement jump performance recovery in the three days following a fatigue protocol involving maximal concentric and eccentric isokinetic contraction, despite daily application of 100 mg of CBD. Similarly, Alpy et al. (2023) found no difference in the recovery of maximal voluntary contraction of the biceps brachii at 30° and 90° elbow flexion angles over 72 h, despite daily application of a 2% CBD ointment.

CBD has previously demonstrated protective effects against muscle degeneration, as observed in Duchenne muscular dystrophy, by reducing inflammation and modulating autophagy (Iannotti et al. 2019). CBD may exert similar protective effects on muscle tissue through activation of the adenosine A2A receptor, which has been shown to reduce markers of muscle damage, such as serum CK activity, in murine models of muscle injury induced by hindlimb ischemia–reperfusion (Alpy 2023). Since CBD can increase extracellular adenosine availability for receptor activation (Schouten et al. 2022), it could theoretically provide tissue-protective benefits during and after intense exercise. However, no evidence of such protective effects was observed in our study. Blood Myo concentration, a primary biomarker of muscle damage (Pastina 2024), remained unaffected by topical CBD application. This result confirms the lack of effectiveness of CBD gel application on muscle function recovery.

For DOMS and despite the potential desensitization effect on TRPV1 receptors involved in this type of pain, no significant effect was observed following application of the gel (Gamelin et al. 2020, Bisogno 2001). This is consistent with the findings of Alpy et al. (2023), who reported no difference in perceived muscle soreness between an arm treated with 2% CBD ointment and the other arm treated with placebo over 3 days following eccentric upper arm exercise. Similarly, Pastina et al. (2024) found no significant change in pressure-pain threshold, measured with a digital pressure algometer, during the three days following a fatigue protocol, despite daily application of 100 mg of CBD compared to placebo. It appears that the effects of CBD depend on the type of pain, as well as on the duration of the pain (acute vs. chronic) and the treatment. In a well-established acute pain model with intradermal electrical stimulation, CBD did not improve pain in healthy subjects, whereas it may alleviate pain in patients receiving regular treatment for chronic pain (Schneider 2022). Moreover, CBD is often combined with THC for analgesic benefits (Villanueva 2022) However, this strategy is not applicable in sports settings due to restrictions imposed by the WADA. It would be worthwhile to explore the use of CBD in conjunction with other compounds compliant with WADA regulations for DOMS prevention.

Thus, the absence of condition differences in muscle damage markers following exercise, as confirmed by previous studies (Alpy 2023; Pastina 2024), suggests that topical CBD application has neither beneficial nor detrimental effects on muscle function recovery. Despite numerous preclinical studies (Gamelin et al. 2020; Schouten et al. 2022) suggesting that CBD is a promising molecule for promoting muscle function recovery and alleviating DOMS following strenuous exercise, its clinical efficiency remains inconclusive (Bezuglov et al. 2024). This lack of effect could be also explained by the dose applied on the skin and the bioavailability of CBD, which depends in part on the route of administration and the type of product used (Schneider 2022). In our study, consistent with other reports on topical CBD applications, the treatment likely enhanced local bioavailability at the skin level (Filipiuc et al. 2023) rather than systemic, as it could be observed with oral administration (Scholfield 2022). However, when considering other studies that used oral administration with doses exceeding 150 mg and thus likely increasing its bioavailability, none reported an effect on the recovery of isometric and isokinetic strength levels, Myo, Interleukine-6 concentrations, or DOMS (Isenmann et al. 2021, 2024). To our knowledge, only Isenmann et al. (2024) have reported a significant beneficial effect of a single oral dose of CBD (60 mg CBD solubilisate in 250 ml of water) on muscle function recovery (squat performance) and on indirect markers of muscle damage (specifically CK and Myo) following strenuous exercise. They also reported similar beneficial effects on Myo following a six-day period of intensive training with a daily intake of CBD-oil (60 mg) in advanced athletes (Isenmann et al. 2024). Interestingly, this protective effect was not observed in highly trained athletes, suggesting that the effects of CBD may be also dependent on training status and bioavailability. It should also be kept in mind that there is a minimum effective dose, which depends on the type of effect being investigated or targeted (Millar et al. 2019). It is likely that the minimum dose of CBD required to exert an effect on muscle damage has not yet been achieved in the available studies and that this dose may vary depending on the parameter investigated (strength level, Myo, or DOMS).

### Limits

Our study has several limitations, including a small sample size and the absence of an a priori power analysis, which means the study is likely underpowered and should therefore be considered a pilot study. In addition, the washout period was too short to prevent the RBE. Another important limitation is the absence of CBD and metabolite quantification in the blood. In our study, a daily dose of 20 mg of CBD was applied topically, which mirrors the relatively low concentrations found in most commercially available creams and gels, potentially limiting bioavailability and, consequently, treatment efficacy (Calvo-Rubio et al. 2024). It remains unclear whether the CBD administered via this route reached the muscle tissue or entered the bloodstream. Indeed, cutaneous application of CBD appears to result in high variability in systemic bioavailability depending not only on the dose but also on the presence of permeability enhancers (Scholfield 2022). In our study, CBD was incorporated into a Carbopol® 980 polymer without the use of penetration enhancers. Furthermore, we analyzed three blood samples at the end of the CBD treatment period, and no trace of CBD was detected. The analyses were not performed for all participants due to limited resources. It is necessary for future studies to include this parameter in order to correlate it with potential effects on recovery markers.

## Conclusion

The findings of this study do not support the effectiveness of topical CBD application in enhancing muscle function recovery or reducing DOMS following strenuous exercise. The only notable difference in isometric peak torque in CBD condition is likely attributable to methodological factors such attrition bias and the repeated bout effect, rather than a genuine therapeutic effect of CBD. These results are consistent with previous studies investigating topical CBD application for muscle recovery (Alpy 2023; Pastina 2024), despite promising preclinical evidence and plausible theoretical mechanisms (Gamelin et al. 2020). Given that CBD bioavailability depends on the route of administration and the formulation used, it is crucial for future studies to ensure appropriate control of both local and systemic bioavailability. However, this still allows athletes to use CBD application for recovery without fearing any impairment in performance. In any case, athletes must remain vigilant regarding the products they consume, which should contain exclusively pure CBD and not broad- or full-spectrum formulations that may include other cannabinoids prohibited by the WADA. Daily use of a broad-spectrum CBD supplement resulted in detectable urinary concentrations of WADA-prohibited cannabinoids in urine (Gillham 2025).

## Supplementary Information

Supplementary Material 1.

## Data Availability

The data that support the findings of this study are not publicly available due to their containing information that could compromise the privacy of research participants, but are available from the corresponding author, F-X. G (laboratory URePSSS – ULR7369), at **francois-xavier.gamelin@univ-lille.fr**, upon reasonable request.

## Abbreviations

CBD: Cannabidiol
Δ9-THC: Δ9-Tetrahydrocannabinol
WADA: World Anti-Doping Agency
DOMS: Delayed-onset muscle soreness
CK: Creatine kinase
Myo: Myoglobin
VAS: Visual analogue scale
RBE: Repeated bout effect
