# Supplementary material for: Topical application of cannabidiol for muscle recovery after exercise-induced muscle damage: a randomized, double-blinded pilot study
Source: J Cannabis Res. 2026 Mar 25;8:63. doi: 10.1186/s42238-026-00420-0 (PMC13137573; doi:10.1186/s42238-026-00420-0)
Supplement: Supplementary file 1 — Supplementary Material 1. [file 42238_2026_420_MOESM1_ESM.docx]

| Supplementary Table 1. Two-Way ANOVA outcomes for muscle function, myoglobin and DOMS Over Time and Session | | | | | |
| --- | --- | --- | --- | --- | --- |
| Parameter | **Main effect ANOVA** | | **F Values** | ***P Values*** |  |
| Isometric peak torque 60° *(N.m)* (n=13) | | Time x Session | *F*_(4, 48)_ = 5.083 | 0.0017 |  |
|  | | Session | *F*_(1, 12)_ = 40.44 | 0.0001 |  |
|  | | Time | *F*_(4, 48)_ = 22.70 | 0.0001 |  |
|  | |  |  |  |  |
| Concentric peak torque 60°.s^-1^ *(N.m)* (n=15) | | Time x Session | *F*_(4, 48)_ = 5.012 | 0.0019 |  |
|  | | Session | *F*_(1, 12)_ = 34.63 | 0,0001 |  |
|  | | Time | *F*_(4, 48)_ = 17.31 | 0.0001 |  |
|  | |  |  |  |  |
| Concentration Myo *(Ln)* (n=11) | | Time x Session | *F*_(1.612, 16.12)_ = 2.586 | 0.1143 |  |
|  | | Session | *F*_(1.000, 10.00)_ = 0.8763 | 0.3713 |  |
|  | | Time | *F*_(1.376, 13.76)_ = 1.011 | 0.3597 |  |
|  | |  |  |  |  |
| VAS score *(u.a)* (n=15) | | Time x Session | *F*_(2.149, 30.09)_ = 0.9578 | 0.4007 |  |
|  | | Session | *F*_(1, 14)_ = 16.16 | 0.0013 | |
|  | | Time | *F*_(2.978, 41.69)_ = 11.85 | 0.0001 | |
| The main effects from ANOVA are as follows: *Time* time effect, *Session* session effect (Session 1 or Session 2), × interaction between variables, NS not significant. F-statistic, degrees of freedom, and p-value from analyses of variance used to compare the effects of placebo or CBD gel between session 1 and session 2 on maximal isometric peak torque, isokinetic concentric peak torque, plasma myoglobin concentration, and VAS score. | | | | | |
